# Perceived but not objective measures of neighborhood safety and food environments are associated with longitudinal changes in processing speed among urban older adults

**DOI:** 10.1186/s12877-024-05068-0

**Published:** 2024-06-25

**Authors:** Jinshil Hyun, Gina S. Lovasi, Mindy J. Katz, Carol A. Derby, Richard B. Lipton, Martin J. Sliwinski

**Affiliations:** 1 Saul R. Korey Department of Neurology, Albert Einstein College of Medicine, 1300 Morris Park Avenue, Bronx, NY 10461 USA; 2 Department of Epidemiology and Biostatistics, Drexel University, 3215 Market Street, 2nd Floor, Philadelphia, PA 19104 USA; 3 Department of Epidemiology & Population Health, Albert Einstein College of Medicine, 1300 Morris Park Avenue, Bronx, NY 10461 USA; 4 Department of Human Development and Family Studies and Center for Healthy Aging, The Pennsylvania State University, 402 Biobehavioral Health Building, University Park, PA 16802 USA

**Keywords:** Neighborhood safety, Physical disorder, Aesthetic quality, Food environments, Cognitive aging, Ambulatory cognition, Longitudinal analysis

## Abstract

**Background:**

Although a growing body of literature documents the importance of neighborhood effects on late-life cognition, little is known about the relative strength of objective and subjective neighborhood measures on late-life cognitive changes. This study examined effects of objective and subjective neighborhood measures in three neighborhood domains (neighborhood safety, physical disorder, food environments) on longitudinal changes in processing speed, an early marker of cognitive aging and impairment.

**Methods:**

The analysis sample included 306 community-dwelling older adults enrolled in the Einstein Aging Study (mean age = 77, age range = 70 to 91; female = 67.7%; non-Hispanic White: 45.1%, non-Hispanic Black: 40.9%). Objective and subjective measures of neighborhood included three neighborhood domains (i.e., neighborhood safety, physical disorder, food environments). Processing speed was assessed using a brief Symbol Match task (unit: second), administered on a smartphone device six times a day for 16 days and repeated annually for up to five years. Years from baseline was used as the within-person time index.

**Results:**

Results from mixed effects models showed that subjective neighborhood safety (*β*= -0.028) and subjective availability of healthy foods (*β*= -0.028) were significantly associated with less cognitive slowing over time. When objective and subjective neighborhood measures were simultaneously examined, subjective availability of healthy foods remained significant (*β*= -0.028) after controlling for objective availability of healthy foods. Associations of objective neighborhood crime and physical disorder with processing speed seemed to be confounded by individual-level race and socioeconomic status; after controlling for these confounders, none of objective neighborhood measures showed significant associations with processing speed.

**Conclusion:**

Subjective neighborhood safety and subjective availability of healthy foods, rather than objective measures, were associated with less cognitive slowing over time over a five-year period. Perception of one’s neighborhood may be a more proximal predictor of cognitive health outcomes as it may reflect one’s experiences in the environment. It would be important to improve our understanding of both objective and subjective neighborhood factors to improve cognitive health among older adults.

**Supplementary Information:**

The online version contains supplementary material available at 10.1186/s12877-024-05068-0.

## Introduction

In recent years, there has been growing interest in the effects of neighborhood characteristics on late-life cognitive health. Various social and physical neighborhood factors, including neighborhood safety, physical disorder or aesthetics, social capital, community-level socioeconomic status, food environments, green space, and local resources (e.g., recreation centers, coffee shops) [1–9], have shown significant associations with late-life cognitive and brain health. These lines of work support the ecological model, which posits that dynamic societal and ecological contexts that individuals are exposed to play a role in health-related behaviors and health outcomes [10]. In the cognitive aging field, researchers extended this theory further and proposed that positive social, physical, and built environments are likely to have downstream effects on health-related behaviors (e.g., physical activity, social engagement, better dietary behavior) and cognitive aging eventually [4, 11].

Most of prior studies, which investigated neighborhood effects on late-life cognition, assessed neighborhood characteristics using either objective (i.e., GIS-based) or subjective (i.e., self-reported) measures, but only a few studies compared effects of objective and subjective neighborhood simultaneously [6]. Because individuals even within the same area can experience their environment differently, objective and subjective neighborhood measures may not overlap but instead provide complimentary information regarding individuals’ health outcomes [12, 13]. The present study aims to investigate the role of objective and subjective neighborhood characteristics on longitudinal cognitive change among racially and socioeconomically diverse, urban older adults. Among various neighborhood measures, this study focused on three neighborhood domains that could provide comparable objective and subjective neighborhood measures: neighborhood safety (perceived neighborhood safety vs. objective violent crime statistics), physical disorder or lack of aesthetic quality (perceived vs. objective ratings on physical environments such as conditions of buildings and streets), and food environments (perceived availability of healthy foods vs. objective healthy food stores).

### Associations of objective neighborhood measures with cognition

Objective neighborhood measures in prior studies typically include (i) contextual built environment (e.g., availability of supermarkets) that may enhance cognition [4], (ii) observations from interviewers/auditors that capture the extent of neighborhood characteristics (e.g., signs of physical disorder) [6], or (iii) compositional measures from a publicly available database (e.g., violent crime) [6], some of which use variable reduction techniques to deal with the challenges of multicollinearity among neighborhood characteristics. For these measures, neighborhoods are typically defined using the administrative boundaries (e.g., census tract, zip code tabulation area (ZCTA)) or a certain geographical distance (e.g., buffer area of 250 ~ 1600 m) around a geocoded home location [3].

Prior studies have shown limited and conflicting results on how objectively measured neighborhood safety, physical disorder, and food environments were associated with late-life cognition, and most of them investigated cross-sectional associations [4–6, 11, 14, 15]. For objective neighborhood safety, studies found that living in the census tract with high violent crime was associated with lower levels of global cognition [6] and reduced information processing performance [15]. For objective neighborhood disorder, some studies found that greater neighborhood disorder was associated with lower levels of cognition [6] and faster cognitive decline [16, 17], while others did not find such association [11]. For objective healthy food environments, FangFang and colleagues found that female participants living far from a supermarket had significantly lower levels of global cognition [14]; but other studies did not find significant cross-sectional association between density of neighborhood healthy food stores and cognition [4, 5]. Evidence for long-term cognitive change associated with these neighborhood features is lacking. In addition, objective neighborhood measures may not reflect how differently individuals experience and interact with their neighborhood environments.

### Associations of subjective neighborhood measures with cognition

Subjective neighborhood measures refer to individual-level perceptions of one’s neighborhood quality. While these measures are likely to be influenced by objective neighborhood features, individuals’ perception of their neighborhood may vary even within the same neighborhood area [18]. Perceived neighborhood quality may reflect how individuals are exposed and experience their neighborhood in everyday lives, determine one’s psychological states and behaviors, and therefore may be a more proximal predictor of cognitive health outcomes. For example, self-reported perceptions of immediate food environment may better reflect individuals’ actual use and experiences of local food stores [5]. Thus, subjective neighborhood measures can provide additional information related to individuals’ health beyond that captured by objective measures.

Subjective neighborhood measures have long been utilized in other health-related disciplines [12, 13]. In cognitive aging and dementia fields, however, relatively fewer studies have examined how perceived or subjective neighborhood measures were associated with late-life cognitive health [3, 5, 7, 19, 20]. These studies have mostly examined cross-sectional associations between subjective neighborhood characteristics and cognitive performance. They found that greater perceived safety was associated with lower levels of spatial performance and executive function [7] and composite cognitive scores [19]. Studies also found that higher levels of perceived physical disorder were associated with lower levels of spatial performance [7], worse episodic memory [20], and lower levels of composite cognitive scores [19]. Better perception of healthy food environments were significantly associated with better processing speed and more accurate memory binding performance [5]. Given limited prior work that examined effects of subjective neighborhood measures on long-term cognitive changes, more longitudinal work is needed.

### Associations of objective and subjective neighborhood measures with cognition

Despite growing interests in the neighborhood effects on late-life cognitive health, little research has evaluated relative implications for cognitive health of objective and subjective measures of local neighborhood environments [5, 6, 21, 22]. For neighborhood safety, Lee and Waite [6] found that the effects of objective neighborhood measures (e.g., violent crime) disappeared after controlling for subjective neighborhood measures (e.g., perceived danger) and household measures, while the effects of subjective measures remained significant. For food environments, Hyun and colleagues [5] found that greater subjective availability of healthy foods was cross-sectionally associated with better cognitive performance even after controlling for objective food environment measure [5]. These studies suggest that subjective neighborhood perception, which is more proximal factors to cognitive health, may have a stronger effect on cognition compared to objective neighborhood measures. Findings from Lee and Waite [6] further suggest that perceived neighborhood characteristics may be pathways through which the effects of objective neighborhood characteristics are associated with late-life cognition, which warrant further investigation. Given the lack of prior studies, simultaneous investigation of objective and subjective measures of neighborhood environment is necessary to advance our knowledge on how neighborhood affects cognitive health.

In addition, aforementioned studies [5, 6] were cross-sectional and evidence in longitudinal studies is lacking. Although cross-sectional studies provide a snapshot of cognitive function associated with neighborhood characteristics, it has limitations such as reverse causality, cohort effects, or self-selection (i.e., individuals self-selected into a certain environment) [23]. Longitudinal studies enable direct study of within-person cognitive changes. By investigating the effects of neighborhood on rates of cognitive changes over time, longitudinal studies can help to identify factors that may alter the course of cognitive decline and inform development of intervention strategies [24–26]. In current study, we compared the relative strength of objective and subjective neighborhood measures in relation to levels (i.e., between-person cognitive differences at cross-section) and rates of cognitive decline (i.e., average trajectories of within-person cognitive change).

### Neighborhood and individual-level sociodemographic factors

Although neighborhood environments may shape health-related behaviors and cognitive health outcomes among people living in the areas, neighborhood may also be a reflection of people living there. People may select themselves into or out of a certain neighborhood [23] and individuals’ sociodemographic factors including race/ethnicity, levels of education, occupation, or income may decide where people live or how large local residents perceive their neighborhood is [12, 27]. These individual characteristics (i.e., racial minority, socioeconomic status) themselves are predictors of cognitive outcomes [28, 29], suggesting that sociodemographic factors may be important confounders to adjust in understanding the associations between neighborhood characteristics and cognitive health.

In addition, contextual influences may be particularly pronounced for vulnerable populations (e.g., racially minoritized individuals), as they may face combined threats from both individual- and contextual-level disadvantages [30]. A few studies examined cross-level interactions between race and neighborhood environments [4, 11, 31] to identify sub-groups of populations that are vulnerable to the effect of neighborhood environments. The results were conflicting with some studies showing no interaction between race and neighborhood resources [4], while others found that more neighborhood resources were differentially associated cognitive function by race [11, 31]. Given limited evidence, the current study explored cross-level interactions between race and neighborhood environments on late-life cognition.

### Cognitive assessments from a measurement burst design

In assessing changes of cognitive performance, conventional longitudinal designs typically consisted of repeated single-shot assessments, usually spaced over one to several years [3]. The repeated single-shot assessments may be subject to temporal sampling error and random measurement error. For example, if an individual experiences a “good day” or a “bad day” relative to his/her own typical cognitive performance, it would be hard to capture an individual’s effective level of cognitive performance and true longitudinal change.

Alternatively, we can employ a measurement burst design, which incorporates “bursts” of intensive repeated assessment within a relatively short period of time (e.g., weeks) that are repeated longitudinally, over more widely spaced temporal intervals (e.g., annually) [32]. By aggregating cognitive test scores that were repeatedly assessed within a short period of time (e.g., weeks), the effects of random and systematic within-person variability may be cancelled out and reduce the effects of “good” or “bad” days on estimated performance. With improved precision and reliability, ambulatory cognitive measures are likely to estimate effective level of cognitive performance and increase the ability to detect subtle longitudinal cognitive decline associated with neighborhood characteristics. Advances in smartphone-based cognitive assessments have enabled frequent and repeated cognitive testing in people’s everyday lives [33].

In this study, we used smartphone-based cognitive assessments in a measurement burst study design to detect subtle cognitive decline. We focused on the cognitive measure of processing speed, which is regarded as an elementary cognitive operation that influences the efficiency of more complex cognitive abilities and precedes age-related normative decline in other cognitive abilities as well as pathologic aging [34–37]. The Einstein Aging Study provided the opportunity to examine the associations of objective and subjective neighborhood measures with long-term changes in ambulatory cognition. We hypothesized that positive neighborhood characteristics, including greater neighborhood safety, better aesthetic quality (i.e., less neighborhood physical disorder), and healthy food environments would be associated with better (i.e., faster) performance at baseline and less cognitive slowing over time, with the effects of subjective measures stronger than objective measures of neighborhood. In addition, we explored whether the associations between different neighborhood characteristics and processing speed would vary by race.

## Methods

### Participants

Participants included 306 older adults who were systematically recruited from registered voter lists in the Bronx, NY [38, 39]. The Bronx has the third highest population density behind Manhattan and Brooklyn among five boroughs in New York City (other boroughs include Queens and Staten Island). Bronx is the most racially and socioeconomically diverse borough/county with higher rates of Black individuals (34.1% vs. 9.9 ~ 30% in other boroughs), less people with higher levels of education (people having at least Bachelor’s degree: Bronx: 21.2% vs. 35.3 ~ 62.8% in other boroughs), higher rates of poverty (26.9% vs. 10.4 ~ 19.0% in other boroughs), and higher rates of food insecurity (18.5% vs. 9.4 ~ 13.9% in other boroughs) [40]. Eligible participants were aged 70 and older, ambulatory, fluent in English, and residents of Bronx County, NY. Exclusion criteria included significant hearing or vision loss, current substance abuse, severe psychiatric symptoms that may interfere with testing, chronic medicinal use of opioids or glucocorticoids, treatment for cancer within the last 12 months, or a diagnosis of dementia at enrollment.

### Procedure

In the recruitment phase, introductory letters were mailed to individuals from sampling frames generated from voter registration lists. A research assistant followed up with a phone call to establish eligibility and schedule a clinic visit. At the baseline clinic visit, written consent was obtained, and participants completed a conventional neuropsychological battery and questionnaires about demographics, medical history, family history, and other socio-behavioral factors. Then participants were given surveys assessing subjective neighborhood quality and other psychosocial characteristics to complete at home and return on their next visit. Participants returned to the clinic site to be trained on the use of study smartphone in which surveys for ecological momentary assessment (EMA) were administered. Written informed consent for the EMA protocol was obtained in this initial clinic visit. On the second clinic visit, participants conducted a 16-day EMA protocol. Each day, participants completed a brief smartphone morning survey upon waking, beeped surveys at 4 quasi-random times during a day, and a bedtime survey (total 6 sessions (i.e., assessments) per day). The smartphone survey included items regarding psychosocial states and several brief ambulatory cognitive tasks including Symbol Match task. This burst assessment protocol was repeated every year. On average, the interval between consecutive bursts was 1.04 years. As the Einstein Aging Study was the on-going longitudinal study since May 2017, the current analysis focused on data frozen as of December 2022. Sample sizes were 306 (Burst 1), 204 (Burst 2), 190 (Burst 3), 144 (Burst 4), and 71 (Burst 5) respectively. The compliance rates across bursts were stable, ranging between 82% and 85%. The Institutional Review Board of the Albert Einstein College of Medicine approved the study protocol and all participants provided informed consent.

### Measures

#### Processing speed

The Symbol Match task was administered on the smartphone to measure cognitive performance on processing speed. Participants were asked to compare three symbol pairs at the top of the screen with two symbol pairs at the bottom of the screen and decide as quickly and accurately as possible which of the bottom-screen pairs matches a top-screen pair. The task comprised of 11 trials for each assessment and each assessment occurred 6 times each day for 16 days. Performance in this task improved across sessions within each burst because of the benefit of practice effects [41]. Prior studies have documented that this task administered on the smartphone in uncontrolled naturalistic settings showed excellent between-person reliability, exhibited construct validity from its correlations with conventional, in-lab assessments, and was feasible among a diverse lifespan sample [33, 42]. For the present study, median scores for response times (unit: seconds) of correct trials were calculated at the session level, and then averaged at each burst level (i.e., Burst 1 to Burst 5) to serve as the dependent variable. Higher values reflected slower processing speed [33].

#### Neighborhood characteristics

We assessed objective and subjective neighborhood measures from three domains including neighborhood safety, physical disorder, and food environment.

*Objective neighborhood measures* Participants’ addresses at baseline were linked to objective neighborhood scores listed below through 11-digit Federal Information Processing System (FIPS) codes.

*Violent crime* was defined as the sum of four offenses that involve force or threat of force, including murder and nonnegligent manslaughter, rape, robbery, and aggravated assault [43, 44]. The scores were summarized at the census tract level and natural log-transformed due to the presence of skewness. The final variable was reverse coded so that higher scores indicated low violent crime.

To assess *neighborhood physical disorder,* we used data from virtual audits of Google Street View imagery, which were generated from the Systematic Transportation and Recreation Environment Evaluations Using Technology (STREET) Project [45]. Trained auditors virtually navigated street segments within the Street View interface in the Computer Assisted Neighborhood Visual Assessment System (CANVAS) and rated neighborhoods based on variables of interest including building condition, building vacancy, broken windows, graffiti, the condition of electrical wires, and litter. Neighborhood physical disorder was the sum of the above mentioned items and aggregated at the census tract level (possible range = 0 to 24) [45]. The final variable was reverse coded so that higher scores indicated low neighborhood physical disorder.

*Healthy food stores* were defined as the density of grocery stores and supermarkets (NAICS 445,110) as well as specialty food stores (NAICS 4452). Establishment variables were derived from the National Establishment Time-Series (NETS) database, and all measures were counts per 1000 population in a census track. To deal with outliers, scores were top-coded to the 99th-percentile [4]. The data for each census tract was obtained from the National Neighborhood Data Archive database [46].

*Subjective neighborhood measures* The current study used perceived neighborhood quality measures (1 = Strongly disagree to 5 = Strongly agree) assessed at baseline [47].

*Perceived neighborhood safety* was measured using three items: “I feel safe walking in my neighborhood at night”, “Violence is not a problem in my neighborhood”, and “My neighborhood is safe from crime”. *Perceived aesthetic quality* was measured using the five items including: “There is a lot of trash and litter in my neighborhood (reverse coded)”, “There is a lot of noise in my neighborhood (reverse coded)”, “In my neighborhood the buildings and homes are well-maintained”, “The buildings and houses in my neighborhood are interesting”, and “My neighborhood is attractive”. *Perceived availability of healthy foods* was measured using the three items including: “A large selection of fresh fruits and vegetables is available in my neighborhood”, “The fresh fruits and vegetables in my neighborhood are of high quality”, and “A large selection of low-fat products is available in my neighborhood”.

Summary scores of each subjective neighborhood measure, as well as scores for each objective neighborhood measure, were Z-scored. For all neighborhood measures, higher scores indicated better neighborhood quality.

#### Covariates

The following covariates were included in the analytic models. *Baseline age* was coded in years (centered at the sample mean), *sex* was coded as ‘male’(reference) and ‘female’, and *race/ethnicity* was coded as ‘non-Hispanic Whites’(reference), ‘non-Hispanic Blacks’, and ‘other race’. *Education* was measured using the highest degree earned and coded as ‘Less than high school completion’, ‘High school diploma/GED’(reference), ‘Associates/Bachelors’, and ‘Masters/Doctorates’. *Retirement status* was coded as ‘retired (1)’ and not ‘retired (0)’. *Financial situation* was assessed at baseline using one item, “How would you rate your financial situation these days?” (0 = Worst possible situation through, 10 = Best possible situation), and Z-scored. We included *the total number of sessions* for each burst to control for retest-related effects.

### Analytic approach

Mixed effects models using SAS PROC MIXED (version 9.4) were used to account for the nested structure of the data (i.e., bursts within persons) using a random intercept for person and a random slope for time. Full maximum likelihood was used for model estimation and robust standard errors were used for fixed effects hypothesis testing. Levels and rates of change in processing speed (i.e., response time in seconds) were modeled as a function of neighborhood measures and a within-person time index (i.e., time from baseline, in years). For all neighborhood variables, higher scores indicated better neighborhood characteristics. Covariates were baseline age, sex, education, race/ethnicity, retirement status, financial situation, and the number of sessions at each burst. See Supplementary Eq. 1 for details.

To examine effects of neighborhood measures on processing speed, models were sequentially constructed. To examine potential confounding effects of race and socioeconomic status (i.e., education, financial situation, and retirement status), Model 1 did not include race and socioeconomic status as covariates and it only included age, sex, number of sessions, and either objective or subjective neighborhood measure from each neighborhood domain. Model 2 further added race and socioeconomic status with either objective or subjective neighborhood measure. Model 3 further included objective and subjective neighborhood measures simultaneously from the same neighborhood domain. As all neighborhood measures were Z-standardized (mean = 0, SD = 1), we would be able to compare relative strength of the effects of each objective and subjective neighborhood measures on processing speed.

To examine whether the associations between neighborhood characteristics and processing speed varied by individual-level sociodemographic factors, the above Model 2 further included interaction terms of neighborhood × race and neighborhood × time × race.

## Results

### Descriptive statistics

Table 1 shows descriptive statistics. Mean age was 77.5 (range = 70 to 91) and women made up 67.7% of the sample. The sample was diverse in terms of race (non-Hispanic White: 45.1%, non-Hispanic Black: 40.9%, Hispanic: 14.1%) and education (Less than high school: 5.9%, High school or GED: 43.1%, Associate: 5.9%, Bachelors: 20.9%, Masters: 18.0%, and Doctorate: 6.2%). Mean follow-up was 3 years for those who had at least one follow-up (range = 0 to 4.9 years).

**Table 1:** Sample characteristics at baseline (Mean (SD) or %)

Variable	All (*N* = 306)
Age	77.47 (4.81)
Female	67.65%
Race	
Non-Hispanic Whites	45.10%
Non-Hispanic Blacks	40.85%
Other race	14.05%
Highest degree earned	
Below high school completion	5.88%
High school diploma/GED	43.14%
Associate	5.88%
Bachelors	20.92%
Masters	17.97%
Doctorate	6.21%
Financial situation	7.08 (2.14)
Retired	91.18%
***Objective neighborhood measure***	
Violent crime	56.04 (60.82)
Neighborhood disorder	2.16 (0.6)
Healthy food stores	1.08 (0.99)
***Subjective neighborhood measure***	
Perceived safety	3.55 (0.98)
Perceived aesthetic quality	3.89 (0.76)
Perceived availability of healthy foods	3.91 (0.83)
***Cognitive performance***	
Symbol Match (response time: seconds)	3.27 (1.38)

*Note* Original scores before transformation were used for descriptive statistics

Result from the correlation analyses (Table 2) exhibited that most objective and subjective neighborhood measures had weak to moderate correlations with each other, suggesting that objective and subjective neighborhood measures were not entirely overlapping and complemented each other in providing information on neighborhood environments. Interestingly, the correlations between objective and subjective neighborhood measures within the same domain were not necessarily stronger than other correlations between objective and subjective neighborhood pairs (see italicized numbers in Table 2). Most neighborhood measures were correlated with each other in the expected direction except for the measure of objective healthy food stores. Greater density of healthy food stores was associated with higher levels of violent crime, lower levels of perceived aesthetic quality, and lower levels of perceived availability of healthy foods. Racial minorities (non-Hispanic Blacks, other race), compared to non-Hispanic Whites, were more likely to live in areas with higher violent crime and greater neighborhood disorder, and perceived their neighborhood less safe, having less aesthetic quality, and lacking healthy food stores (Supplementary Table 1).

**Table 2:** Correlations among variables of interest at baseline

	1	2	3	4	5
***Objective measures***					
1. Low violent crime	1.00				
2. Low neighborhood disorder	**0.25** ^*^	1.00			
3. Healthy food stores	**-0.13** ^*^	-0.05	1.00		
***Subjective measures***					
4. Perceived safety	***0.40*** ^*^	**0.23** ^*^	-0.10	1.00	
5. Perceived aesthetic quality	**0.34** ^*^	***0.30*** ^*^	**-0.23** ^*^	**0.65** ^*^	1.00
6. Perceived availability of healthy foods	**0.26** ^*^	**0.21** ^*^	***-0.14*** ^*^	**0.55** ^*^	**0.58** ^*^

*Note* Spearman correlation analyses were conducted; ^*^p < 0.05. Objective violent crime and objective disorder were reverse coded so that higher scores indicated better neighborhood characteristics for all neighborhood measures. Italicized numbers indicate correlations between subjective and objective measures in the same domain

### Neighborhood measures and cognitive trajectories

We first fit the covariates-only model to examine the effects of covariates on cognition (Supplementary Table 2). The results from the mixed model indicated that response time was slower among older participants and non-Hispanic Blacks. The number of sessions at each burst was significant, indicating significant retest-related improvement in cognitive performance. Linear and quadratic time effects showed accelerated slowing in response time across 5 bursts. The random effect for time indicated significant inter-individual variation in rates of cognitive change. All covariates, random effect of time, and autocorrelation term (AR(1)) were included in subsequent analyses.

We tested whether measures of objective and subjective neighborhood were associated with baseline levels of processing speed (measured in response time) and rates of change in processing speed over time (Table 3). For neighborhood safety domain, lower level of objective violent crime was significantly associated with faster response time at baseline when race and socioeconomic status (education, financial status, retirement status) were not controlled for (Model 1: *β* = -0.191). When these sociodemographic factors were entered (Model 2), objective violent crime was not significantly associated with levels of processing speed anymore. Together, results from Models 1 and 2 showed confounding effects of sociodemographic factors in the association between objective crime and initial levels of processing speed. For rates of change, the effects of objective crime on rates of processing speed were not significant in any models. For subjective measures of safety, perceived safety was significantly associated with faster response time at baseline when sociodemographic confounders were not controlled for (Model 1, *β* = -0.190). When these confounders were controlled in Model 2, the effect became non-significant. With rates of change, perceived safety showed significant associations with less slowing in speed over time even after controlling for confounders including race and socioeconomic status (Model 2: *β* = -0.028; 1 SD increase in perceived safety was associated with less slowing in response time by 28 milliseconds per year). When objective crime was controlled for in Model 3, the effect of perceived safety was somewhat attenuated and marginally significant (*β* = -0.025, *p* = 0.054).

**Table 3:** Effects of objective and subjective neighborhood on processing speed (standardized coefficients (standard error)) (unit: seconds)

Neighborhood domain	Neighborhood measure	Separate Model ^a^			Simultaneous Model ^b^
		Model 1: Without race and SES	Model 2		Model 3
Safety	Objective violent crime (reverse) ^c^	**-0.191** **(0.057)** ^*^	-0.069 (0.118)		-0.024 (0.130)
	Objective violent crime (reverse) × Time	-0.011 (0.012)	-0.023 (0.019)		-0.015 (0.019)
	Perceived safety	**-0.190** **(0.073)** ^*^	-0.115 (0.063)^+^		-0.110 (0.074)
	Perceived safety × Time	-0.022 (0.012)^+^	**-0.028** **(0.013)** ^*^		-0.025 (0.013)^+^
Physical disorder	Objective disorder (reverse) ^c^	**-0.130** **(0.06)** ^*^	-0.057 (0.072)		-0.072 (0.065)
	Objective disorder (reverse) × Time	-0.016 (0.015)	-0.016 (0.016)		-0.010 (0.016)
	Perceived aesthetic quality	-0.031 (0.084)	0.066 (0.118)		0.084 (0.116)
	Perceived aesthetic quality × Time	-0.020 (0.013)	-0.023 (0.014)		-0.021 (0.014)
Food environments	Objective healthy food stores	-0.052 (0.047)	-0.078 (0.05)		-0.085 (0.047)^+^
	Objective healthy food stores × Time	0.003 (0.009)	0.002 (0.009)		0.002 (0.009)
	Perceived availability of healthy foods	**-0.171** **(0.049)** ^*^	**-0.116** **(0.054)** ^*^		**-0.119** **(0.053)** ^*^
	Perceived availability of healthy foods × Time	-0.023 (0.013)^+^	**-0.028** **(0.013)** ^*^		**-0.028** **(0.013)** ^*^

^*^p < 0.05, ^+^*p* < 0.10;

^a^ Either objective or subjective neighborhood measure included

^b^ Both objective or subjective neighborhood measure included; Model 1 controlled for age, sex, and number of sessions. Model 2 further controlled for race, education, retirement status, and financial situation

^c^ Objective violent crime and objective disorder were reverse coded so that higher scores indicated better neighborhood characteristics. As all neighborhood measures were Z-scored, 1 unit increase indicates 1 SD increase in each neighborhood measure

For neighborhood physical disorder domain, lower level of objective physical disorder was significantly associated with faster response time at baseline when race and socioeconomic status were not controlled for (Model 1: *β* = -0.130). When potential confounding by sociodemographic factors was controlled in Models 2, significant effect of objective physical disorder on levels of processing speed disappeared. Physical disorder was not associated with rates of change in processing speed in any models. For subjective measure, perceived aesthetic quality was not significantly associated with either levels or rates of change in processing speed in any models.

**Fig. 1:**
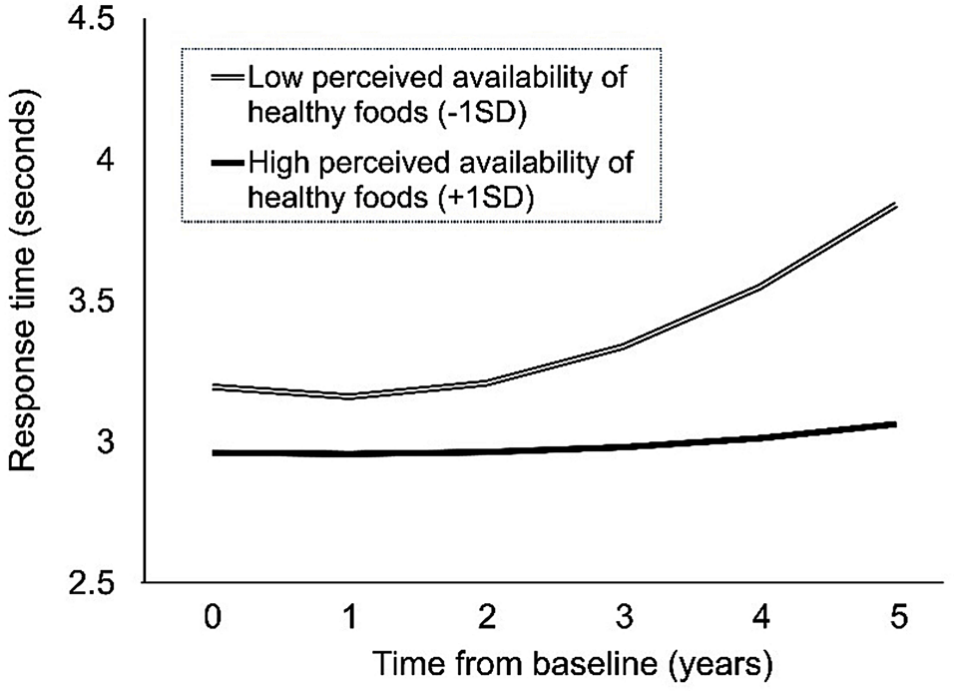
Predicted changes in processing speed between individuals with low and high perceived availability of healthy foods

For neighborhood food environments domain, objective healthy food environments were not associated with levels or rates of change in processing speed. For subjective food environments, greater perceived availability of healthy foods was associated with faster response time at baseline after controlling for individual-level covariates and confounders (Model 2: *β* = -0.116); the significant effect remained after controlling for objective healthy food environments (Model 3: *β* = -0.119). For rates of change, greater perceived availability of healthy foods was associated with less slowing in response time after controlling for individual-level variables and sociodemographic covariates (Model 2: *β* = -0.028) and further controlling for objective food environments (Model 3: *β* = -0.028). Figure 1 illustrates the effect of perceived availability of healthy foods on processing speed over time.

Next, we tested whether the effects of neighborhood varied by individuals’ race. There were no significant interactions between neighborhood × race and neighborhood × time × race with any neighborhood measures.

We conducted several sensitivity analyses. First, to rule out the possibility that individuals who are more physically and mentally healthy rated their neighborhood positively [48], we additionally controlled for baseline depression score and difficulties with physical activities, and the pattern of results did not change. Second, as our financial situation measure was based on self-reports, we conducted analyses using participants’ income or subjective ratings on having difficulties in paying monthly bills. The pattern of results did not change. Third, to examine whether there was evidence of selective attrition by subgroups of populations, we compared differences in follow-up length by subgroups of populations. There were no significant differences in the length of follow-up years by race, education, and each neighborhood measure.

## Discussion

This study examined the effects of objective and subjective measures in three neighborhood domains (i.e., neighborhood safety, physical disorder, and food environments) on long-term changes in processing speed among older adults. The use of ambulatory cognition allowed improved precision and reliability to detect subtle longitudinal cognitive slowing associated with neighborhood characteristics. We found that objective neighborhood measures of violent crime, physical disorder, and healthy food environments were not related to either baseline levels or rates of change in processing speed after controlling for individual and sociodemographic variables. But subjective neighborhood measures including greater perceived safety and perceived availability of healthy foods showed significant effects on better trajectories of processing speed after controlling for individual and sociodemographic factors. These findings indicate that residents’ perceptions of neighborhood environments, compared to objective neighborhood environments, are more proximally related to cognitive health. Given no prior studies on the relative importance of objective and subjective neighborhood measures on long-term cognitive decline, this study importantly extends prior cross-sectional work [5, 6, 21] by demonstrating important role of subjective neighborhood measures on cognitive aging.

The size of correlations between corresponding objective and subjective neighborhood measures was weak to moderate (*rs* = -0.14 ~ 0.40), indicating that objective and subjective neighborhood measures are linked yet distinct constructs and that they can provide complementary information on cognitive health. This finding also suggests that the objective neighborhood environments are not the only source that impacts individuals’ neighborhood perceptions but there are other factors affecting neighborhood perceptions. For example, if individuals were exposed to violent crime, their subjective neighborhood safety ratings may be different from others who were not exposed to crime in the same neighborhood. In addition, objective measures of food environments may not capture important non-geographic dimensions (e.g., transportation, local food stores’ business strategies) that affect individuals’ healthy food perceptions and choices [49, 50]. Future studies will be required to investigate factors influencing perceived neighborhood measures over and above the effects from objective neighborhood factors.

We did not find significant effects of objective neighborhood measures on processing speed. However, caution is required in interpreting non-significant effects of objective neighborhood measures. It does not necessarily suggest that objective neighborhood environments are less important for cognitive health. Rather, it may point to the need for using more fine-grained neighborhood boundaries or person-specific assessments of neighborhood environments. Although studies (including the current study) often rely on census geography to operationalize the neighborhood units [3, 46], these artificial neighborhood units may not reflect heterogenous neighborhood experiences across individuals. Measurement error in neighborhood area based on geospatial coding may attenuate associations between neighborhood measures and outcomes [27]. Use of spatial technologies such as Global Positioning System (GPS) would be able to help future research to better reflect person-specific neighborhood exposure and experiences that are closely related to older adults’ everyday lives and cognitive health [51].

For neighborhood safety domain, perceived safety was associated with less cognitive slowing over time after controlling for individual-level sociodemographic factors. The result extends prior cross-sectional research of Lee and Waite [6] that found stronger effects of subjective safety and demonstrates the important role of perceived safety with long-term cognitive decline. Prior studies have suggested several behavioral and psychological mechanisms in this association [4, 11, 17]. Favorable neighborhood perceptions of safety may enhance health-related behaviors such as exercising and engaging in social activities [52–54], which in turn contribute to better cognitive function and decrease rates of cognitive decline by facilitating neuroplasticity process [55, 56]. In addition, neighborhood environments with better perceived safety may provide easier access to environmental resources that can benefit cognitive stimulation (e.g., better public spaces, greater density of institutional resources) [17]. On the other hand, lack of perceived neighborhood safety may act as chronic stress, which may activate greater emotional and physiological responses in the event of stress [18] and lead to long-term cognitive decline through glucocorticoid related mechanisms [57–59].

For food environments domain, perceived availability of healthy foods was significantly associated with both levels and rates of change in processing speed after controlling for individual-level factors. The effects of perceived availability of healthy foods were also independent of objective food environments. It is likely that perception of better food availability, rather than food environments in local districts, may be closely related to health-related behaviors and better diet. Prior literature suggests that favorable perception on their food environments may promote health-related behaviors such as greater fruit and vegetable consumption [60, 61] and physical activity [62], which can decrease vascular risks and protect against cognitive impairment [5, 56, 63]. In addition, perceived availability of healthy foods may be a proxy for the availability of other institutional resources (e.g., libraries, community centers, shopping places). Older adults living in the area with the better institutional resources may have a lifelong history of living in more resource-rich environments, which might have helped them to accumulate cognitive reserve [11] and resulted in better baseline performance as well as less cognitive decline over time. Alternatively, the result may indicate lack of relevance in the measure of objective food environments that relate to health outcomes. We found unexpected associations between the objective food environment measure and other neighborhood measures such that higher density of objective healthy food stores was related to lower perceived availability of healthy foods, lower perceived aesthetic quality, and higher violent crime. In Bronx NY, areas characterized by low density of food stores included relatively rich residential areas that are located just outside of big supermarket locations; residents are likely to have easy access to those stores by car or via delivery. On the other hand, neighborhood areas having high density of food stores may be characterized as commercial areas that include other stores with adverse effects on health. Then other objective measures such as affordability of foods would better reflect food environments related to health.

For neighborhood physical disorder domain, we did not find significant effects of neighborhood disorder (either objective or subjective) on levels and rates of change in processing speed. Results from previous coordinated analyses [7] also found less consistent effect of perceived neighborhood aesthetic quality than perceived neighborhood safety on levels of cognition. It is possible that, compared to neighborhood physical disorder or lack of aesthetics, neighborhood safety has more direct impact on individuals’ security and survival, activate emotional and physiological stress response, and result in cognitive impairment [58, 64]. However, other studies found significant associations between objective physical disorder and worse decline in global cognition [16, 17]. Given the lack of longitudinal studies examining both objective and subjective physical disorder measures, future studies are needed to examine effects of these measures on different cognitive domains.

We found that individual-level sociodemographic factors such as race and socioeconomic status were important confounders to adjust. Individual-level sociodemographic factors seemed to be intertwined with neighborhood selection; they can make the associations between neighborhood measures and cognition statistically correlated even though there is no direct causal link between them. Future studies will need to identify and adjust important individual-level confounders, investigate cognitive health disparities by sociodemographic and neighborhood factors, and utilize longitudinal study designs that help to identify potential causal mechanisms [65, 66].

We did not find significant interaction between race and neighborhood factors on processing speed. This finding suggests that the protective effects of greater perceived safety and greater perceived availability of healthy foods on processing speed may be similar across racial groups. Given that racial minorities were more likely to live in areas with negative neighborhood features (e.g., lower perceived safety, lower availability of healthy foods), interventions to change perceived neighborhood quality (e.g., organize neighborhood safety efforts; improve quality of supermarkets serving vulnerable populations, include culturally relevant healthy foods) [67, 68] may protect older adults, especially vulnerable populations, from cognitive impairment.

There are some limitations to this study. First, subjective measures of neighborhood may reflect both observations and preferences (e.g., measurable decibels vs. preference in measuring neighborhood noise), but we could not separate them. It will be important to investigate factors that underlie how individuals rate their subjective neighborhood perception (e.g., activity space; neuroticism). Second, future studies need to examine behavioral and psychological mechanisms in the associations between neighborhood and long-term cognitive change in order to identify targetable areas for intervention. Third, although we controlled for perceived financial status and levels of education, there might have been unmeasured confounders that we could not adjust in this study (e.g., wealth). Fourth, this study was conducted in the urban Bronx, NY, which is the ninth-most-populous city/borough in the US. Although the wide variability of neighborhood characteristics allowed us to analyze differential neighborhood risk factors, the study result may not be generalizable to older adults residing elsewhere. Fifth, properly accounting for retest/practice effects has been a challenge in cognitive aging research. In future research, the use of advanced methods [41, 69] may allow us to separate aging-related cognitive decline from retest-related gains so that we can obtain more precise estimations of individual differences in rates of cognitive change. Sixth, this study focused on processing speed, which is considered to precede age-related decline in other domains. Future studies will need to investigate other performance measures in higher-order cognitive domains.

## Conclusion

The present study examined prospective associations of objective and subjective neighborhood measures and changes in processing speed over a five-year period among racially and socioeconomically diverse community dwelling older adults. We found that subjective neighborhood safety and subjective availability of healthy foods, rather than objective measures, were associated with less cognitive slowing over time. Results suggest that perception of one’s neighborhood, rather than objective measures, may be a more proximal predictor of cognitive health outcomes as it may reflect heterogeneous neighborhood exposures and experiences across individuals.

These findings have methodological implications for future research. As ratings on subjective neighborhood perception may provide complementary information on objective and built environment characteristics, both subjective and objective neighborhood measures need to be included to identify neighborhood characteristics linked to cognitive health. In addition, more fine-grained objective neighborhood measures would be required to assess individuals’ experiences and exposures in neighborhood environments (e.g., personalized activity space). Further, public efforts would be required to increase perceived neighborhood quality to promote cognitive health among older residents. As an analysis of an intervention, residents’ perceptions of the environment within a certain neighborhood area may be assessed before and after the implementation of neighborhood change to evaluate the effectiveness of the policy.

### Electronic supplementary material

Below is the link to the electronic supplementary material.

Supplementary Material 1

## Data Availability

The datasets generated and/or analyzed during the current study are available from the corresponding author after obtaining approval from the Einstein Aging Study Scientific Committee.

## Abbreviations

EMA: Ecological momentary assessment
ZCTA: Zip code tabulation area
