# Supplementary material for: Perceived but not objective measures of neighborhood safety and food environments are associated with longitudinal changes in processing speed among urban older adults
Source: BMC Geriatr. 2024 Jun 25;24:551. doi: 10.1186/s12877-024-05068-0 (PMC11197239; doi:10.1186/s12877-024-05068-0)
Supplement: Supplementary file 1 — Supplementary Material 1 [file 12877_2024_5068_MOESM1_ESM.docx]

# Supplementary Materials

Supplementary Equation 1.

Shown below is the multilevel model for the effect of neighborhood measures on Symbol Match task performance (i.e., response time) that specifies two levels of analysis.

Level 1:

$Response time_{ij}=b_{0j}+b_{1j}\left( Linear time_{ij} \right)+b_{2j}\left( Quadratic {time}_{ij} \right)+ b_{3j}\left( Number of Sessions_{ij} \right)+e_{ij}$

The Level 1 model describes within-person variation in response time of the Symbol Match task for person j on Burst i as a function of a person-specific intercept ($b_{0j}$), linear and quadratic time effect ($b_{1j}, b_{2j}$), number of sessions ($b_{3j}$), and an assessment- and person-specific residual deviation from that intercept ($e_{ij}$).

Level 2:

$$b_{0j}= \beta_{00}+\beta_{01}\left( Neighborhood measure_{.j} \right)+\beta_{02}\left( Covariat{es}_{.j} \right)+u_{0j}$$

$$b_{1j}= \beta_{10}+\beta_{11}\left( Neighborhood measure_{.j} \right)+\beta_{12}\left( Covariat{es}_{.j} \right)+u_{1j}$$

$b_{2j}= \beta_{20}$

$$b_{3j}= \beta_{30}$$

The Level 2 model describes between-person variation in the mean response time. $\beta_{00}$ represents the sample average response time at baseline for 77-year-old, non-Hispanic White men with high school diploma who did not retire and had average financial situation rating. $\beta_{01}$ reflects the difference in response time with a 1 unit (i.e., 1 SD) between-person difference in the neighborhood measure. $\beta_{02}$ indicates the difference in response time with a 1-unit difference in each covariate. $\beta_{10}$ indicates average rate of cognitive change over time, and $\beta_{11}$ and $\beta_{12}$ indicate average rates of cognitive change related to neighborhood and covariates respectively. $\beta_{20}$ indicates quadratic time effect and $\beta_{30}$ indicates effects of total session number participants completed per each burst. Finally, $u_{0j}$ and $u_{1j}$ reflect person-specific deviations from the average level and average rate of response time respectively. Our interest was to examine $\beta_{01}$ and $\beta_{11}$, effects of neighborhood measures on levels and rates of change in processing speed after controlling for covariates.

Supplementary Table 1. Neighborhood characteristics by race (Mean (SD)).

| Neighborhood | All |  | non-Hispanic Whites (N=138) | non-Hispanic Black (N=125) | Other race (N=43) | *p-value* |
| --- | --- | --- | --- | --- | --- | --- |
| Violent crime | 56.04 (60.82) |  | 24.02 (38.76) | 89.58 (61.84) | 61.3 (62.69) | 0.000 |
| Neighborhood disorder | 2.16 (0.6) |  | 1.95 (0.46) | 2.26 (0.6) | 2.57 (0.73) | 0.000 |
| Healthy food stores | 1.08 (0.99) |  | 1.01 (0.95) | 1.08 (1.03) | 1.29 (1) | 0.283 |
| Perceived safety | 3.55 (0.98) |  | 3.89 (0.88) | 3.27 (0.99) | 3.27 (0.97) | 0.000 |
| Perceived aesthetic quality | 3.89 (0.76) |  | 4.09 (0.65) | 3.76 (0.76) | 3.61 (0.91) | 0.000 |
| Perceived availability of healthy foods | 3.91 (0.83) |  | 4.12 (0.75) | 3.77 (0.84) | 3.63 (0.89) | 0.000 |

*Note. p*-values were based on regression analyses.

Supplementary Table 2. A covariates-only model predicting processing speed (unit: seconds).

|  | Estimate | SE | *p* |
| --- | --- | --- | --- |
| ***Fixed effects*** |  |  |  |
| Intercept | 3.042 | 0.187 | <.001 |
| Baseline age | 0.029 | 0.012 | 0.011 |
| Sex ^a^ | -0.072 | 0.128 | 0.572 |
| Less than HS ^b^ | 0.415 | 0.315 | 0.188 |
| Associate and BA ^b^ | -0.080 | 0.175 | 0.649 |
| MA and Dr ^b^ | -0.085 | 0.153 | 0.581 |
| Black race ^c^ | 0.383 | 0.154 | 0.014 |
| Other race ^c^ | 0.260 | 0.161 | 0.108 |
| Retired | 0.260 | 0.170 | 0.127 |
| Financial situation | -0.106 | 0.072 | 0.142 |
| Number of sessions | -0.001 | 0.001 | 0.015 |
| Time (in year) | -0.116 | 0.060 | 0.057 |
| Quadratic time | 0.041 | 0.008 | <.001 |
| Baseline age x Time | 0.003 | 0.003 | 0.218 |
| Sex x Time ^a^ | 0.011 | 0.031 | 0.724 |
| Less than HS x Time ^b^ | 0.100 | 0.098 | 0.310 |
| Associate and BA x Time ^b^ | -0.056 | 0.037 | 0.136 |
| MA and Dr x Time ^b^ | -0.076 | 0.047 | 0.105 |
| Black race x Time ^c^ | -0.045 | 0.038 | 0.232 |
| Other race x Time ^c^ | 0.037 | 0.036 | 0.298 |
| Retired x Time | -0.009 | 0.051 | 0.861 |
| Financial situation x Time | 0.013 | 0.018 | 0.461 |
| ***Random effects*** |  |  |  |
| Var (Intercept) | 1.687 |  | <.001 |
| Var (Time) | 0.025 |  | <.001 |
| Cov (Intercept, Time) | 0.037 |  | 0.223 |
| AR(1) | 0.395 |  | 0.003 |
| Residual | 0.089 |  | <.001 |

^a:^ reference: male; ^b:^ reference: high school graduate; ^c:^ reference: non-Hispanic White.

*Note*. Positive estimates indicate slower response time (i.e., worse processing speed), and negative estimates indicate faster response time (i.e., better processing speed).
